# Causes of short stature identified in children presenting at a tertiary care hospital in Multan Pakistan

**DOI:** 10.12669/pjms.291.2688

**Published:** 2013

**Authors:** Muhammad Waqar Rabbani, Waqas Imran Khan, Ahmad Bilal Afzal, Waqas Rabbani

**Affiliations:** 1 Dr. Muhammad Waqar Rabbani, DCH, FCPS, Department of Pediatric Medicine, The Children Hospital & The Institute of Child Health, Multan, Pakistan.; 2 Dr. Waqas Imran Khan, DCH, FCPS, Department of Pediatric Medicine, The Children Hospital & The Institute of Child Health, Multan, Pakistan.; 3 Dr. Ahmad Bilal Afzal, MBBS, Department of Pediatric Medicine, The Children Hospital & The Institute of Child Health, Multan, Pakistan.; 4 Dr. Waqas Rabbani, MBBS, Dept. of Behavioral Sciences, University of Health Sciences Lahore.

**Keywords:** Short stature, Constitutional delayed growth and maturation (CDGM), Familial short stature (FSS), Normal variant short stature (NVSS), Growth hormone deficiency (GHD)

## Abstract

***Objective: ***To determine the frequency of common causes of short stature in children presenting to the Children’s Hospital & the Institute of Child Health, Multan.

***Methodology: ***This cross sectional study was done in Pediatric Endocrinology department, the Children’s Hospital & the Institute of Child Health, Multan, from March to September, 2011. One hundred and sixty nine children with short stature presenting to the outpatient department meeting inclusion criteria were recruited after taking an informed consent. The detailed history, physical examination including anthropometric measurements and relevant investigations were recorded. Causes of short stature (outcome variable) were recorded on a predesigned proforma for final analysis.

***Results: ***The common causes of short stature identified were; familial short stature (FSS) 36 cases (21.3%), hypothyroidism 29(17.2%), growth hormone deficiency (GHD) 18(10.7%), insulin dependent diabetes mellitus (IDDM) 16(9.5%) and constitutional delayed growth and maturation (CDGM) 11(6.5%) cases. This was followed by primary malnutrition 8(4.7%), celiac disease 6(3.6%),Turner syndrome 5(3%) cases and unknown syndromes 4(2.4%) followed by other rare causes.

***Conclusion: ***Common causes of short stature identified in this study were endocrine diseases followed by normal variant short stature (NVSS), while nonendocrine causes were the least.

## Introduction

 Growth is a continuous biologic process subject to genetic, environmental, nutritional and hormonal influences. Altered growth potential may result from disturbance of any of these factors. Short stature, a common problem in child population of developing countries,^1^ is defined by height or length below 3^rd^ centile or less than 2 standard deviation for that specific age and sex.^2^ There is a diverse range of causes of short stature, but fortunately, the normal variant short stature do not need any medical or hormonal treatment, however, associated emotional stress should be addressed appropriately.^3^

 Chronic childhood diseases, if sufficiently severe, can lead to growth failure and short stature, important examples include; renal, pulmonary and cardiac diseases, malignancy, cystic fibrosis and celiac disease.^4^^-^^6^ Celiac disease is a prominent example of treatable causes of growth failure especially in young children.^6^ Malnutrition and iatrogenic causes like glucocorticoids, chemotherapy and radiotherapy are other important causes.^7^ Common endocrine disorders leading to short stature include; hypothyroidism, cushing’s syndrome and growth hormone deficiency.^4^^,^^8^ Turner’s syndrome and skeletal dysplasias, like achondroplasia, are other notable causes. Growth failure is also seen with symmetric intrauterine growth retardation (IUGR) leading to small for gestational age (SGA) babies.^1^^,^^2^^,^^9^ Idiopathic short stature is, quite understandably, a diagnosis of exclusion.^10^

 The final adult height in humans is controlled by multiple genes. In familial short stature, the final adult height is short but within the target range of height for the family.^11^ Constitutional delay of growth and maturation (CDGM) having subtle defects in growth hormone-insulin like growth factor (GH-IGF) axis, obligates higher rates of overall energy expenditure compared with age and size matched controls, this increased metabolism may result in impaired tempo of growth.^12^ Puberty is delayed and there is a delayed and reduced pubertal growth spurt, but the final adult height is usually not affected and remains in the lower parental target height zone.^13^ Those boys whose height predictions fall below160 centimeters, are candidates for treatment with recombinant growth hormone (rGH) according to FDA-approved guidelines is under investigation.^14^

 Endocrine causes are classically associated with being overweight for height. In appropriately treated children with growth hormone deficiency or congenital hypothyroidism, puberty and final adult height are within the normal range in treated cases.^15^ Severe malnutrition (3^rd^ degree) is one of the common causes of short stature in third world countries. The nutritional status of children under five years of age is extremely poor in Pakistan, about 40% children under five years old are short statured and will often have lifelong consequences, with diminished skeletal growth.^16^ Specific nutritional deficiencies can have an effect on child growth. Vitamin D deficiency is an important cause of short stature in third world. The typical infant is exclusively breastfed and has poor exposure to the sun. The treatment regimen improves rickets but does not necessarily correct growth failure, starting treatment in early infancy leads to greater final adult height.^17^

## Methodology

 Study was conducted at the Paediatric Endocrinology Department, the Children’s Hospital and the Institute of Child Health, Multan; a tertiary care pediatric center, from March to September 2011. Children from both genders, 2-15 years of age having height below two standard deviation (-2SD) or less than 3^rd^ percentile for age and sex were recruited. Patients with contractures and kyphoscoliosis in whom height could not be measured were excluded. It was a nonprobability consecutive sampling and sample size was calculated with reference to a prospective study done by Moayeri H et al at Iran,^18^ taking p=0.076 (known proportion for hypothyroidism which was 7.6%),d=0.04 (required absolute precision which was considered 4%) and confidence level of 95% using a software “S size for estimation of population propotion, developed by KC Lun and PeterChaim from National University of Singapore”.

 Protocol of the study was approved by the institutional ethical committee. After explaining all the details of the study, informed consent was taken from the parents/guardians. All the related terminologies were clearly defined.

***Short Stature: ***Height below 2SD or 3^rd^ percentile for age and sex.

***NVSS (Normal variant short stature): ***They include, Constitutional delayed growth and maturation (CDGM) and Familial short stature (FSS).

***Constitutional Delayed Growth and Maturation: ***Bone age equal to height age, both less than chronological age.

***Familial Short Stature: ***Bone age equal to chronological age and both more than height age. ***Growth Hormone Deficiency *(GHD): **Levels less than 10ng/ml on insulin stress test.

***Primary Malnutrition*** **(3** ^rd^ **Degree):** Weight less than 60% of the expected weight for age and sex according to NCHS (National Child Health Services) standard and nutritional history of decreased caloric intake.

***Celiac disease: ***Duodenal mucosal changes consistent with Celiac Disease on small intestinal biopsy and raised tissue transglutaminase antibodies i.e. IgA above 7U/ml and IgG above 17U/ml.

***Hypothyroidism: ***Free T4 less than 0.93ng/dL and TSH more than 6.4 uIU/ml.

***Genetic Syndromes:*** Like (Down syndrome, Turner’s syndrome, Noonan’s syndrome, Russel Silver syndrome) and ***Chronic Diseases*** like (chronic kidney disease, insulin dependent diabetes mellitus, bronchial asthma) were diagnosed from clinical profile and relevant investigations.

 The demographic profile, detailed history including; history of low birth weight (less than 2.5kg), psychosocial aspects and physical examination findings were recorded. This included anthropometric measurements of the patients and their parents. Contemporary height was measured without shoes or head gear on an accurate measuring device (Harpenden Stadiometer). The target height was calculated based on the Tanner’s method^19^ using following formula: [father’s height (cm) + (mother’s height (cm) + 13)] divided by 2 for boys and [(father’s height(cm) – 13) + mother’s height (cm)] divided by 2 for girls. Most children attain an adult height within 10 cm of their target height which provides a quick and rationally precise index of a child’s genetic growth potential. Lower segment was calculated by subtracing sitting height from standing height, upper to lower segment ratio (US/LS) was calculated from these measurements. Weight of the patients was measured using electronic balance and recorded in decimal of kilogram. Puberty was assessed in 11-15 year age group by rating the breast development in girls, genital developments in boys, pubic and axillary hair development in both sexes, according to Tanner′s classification.^20^^,^^21^ Parents were asked for any available previous record.

 Laboratory investigations included (complete blood count, ESR, urinalysis, hepatic and renal parameters, bone profile, Anti tissue transglut aminase (Anti-tTG IgA & IgG), serum free T4 and TSH levels). The patients who had raised levels of Anti-tTGs were confirmed with endoscopic duodenal biopsy. Radiograph of left hand and wrist were done in all patients for rickets and bone age estimation using published standards of Greulich and Pyle’s Atlas of Skeletal Development.

 Short statured patients with chronic diseases were diagnosed on the basis of history, physical examination and relevant investigations. Patients who had weight less than 60% of the expected for age and sex according to NCHS (National Child Health Services) standard with history of deficient caloric intake, evidence of other micronutrient deficiencies and absence of any chronic disease, were labeled as cases of Primary malnutrition (3^rd^ degree). Karyotyping was done in all female patients to rule out Turner Syndrome. GH was assessed (provocating with insulin tolerance test)in those patients who had strong clinical suspicion of growth hormone(GH) deficiency having baseline investigations within normal limits. Blood samples were drawn at 0, 30, 60, 90 and 120 minutes. The final cause of short stature was decided in consultation with the Paediatric endocrinologist.

 Data was entered in SPSS version (10.0). Descriptive statistics were applied. Mean and standard deviation for age were computed. Frequency of various causes of short stature (CDGM, FSS, GHD, IDDM, primary malnutrition, celiac disease, hypothyroidism, genetic syndromes, chronic diseases) was calculated. Confounding variables like age and gender were controlled by stratification.

## Results

 A total of 169 cases [91 males (53.8%), 78 females (46.2%)] were identified as having short stature; male to female ratio was 1.17:1. All children fell below 3rd centile for their height on 2000 CDC growth charts. Among the study population, height of the patients ranged from 75.2-98cm in 2-5 years, 85-126cm in >5-11 and 112-151cm in >11-15 years of age groups.

 There were 40 (23.7%) children having age between 2-5 years, 82 (48.5%) children were >5-11years of age, while the rest of 47 (27.8%) cases were >11 to 15 years of age (Table-I). Family history of short stature was present in 46 (27.2%) children while history of constitutional delayed growth in13 (7.7%).

**Table-I:** Age Distribution of the Children with Short Stature (n = 169).

Age (in years)	No. of Children	Percentage (%)
2-5	40	23.7
>5–11	82	48.5
>11 – 15	47	27.8
Total	169	100.0

Note: Age of the children has been rounded to nearest year.

Key: Age range = 2 – 15 years.

**Table-II:** Causes of Short Stature in Relation to Gender Distribution (n = 169).

*Causes*	*Total No. of Children*	*No. (%) of male Children*	*No.(%) of female Children*
Constitutional growth delay	11(6.6%)	7(63.6%)	4(36.4%)
Familial short stature	36(21.3)	22(61.1%)	14(38.9%)
Growth hormone deficiency	18(10.7%)	8(44.4%)	10(55.6%)
Primary malnutrition	8(4.7%)	3(37.5%)	5(62.5%)
Celiac disease	6(3.6%)	2(33.3%)	4(66.7%)
Hypothyroidism	29(17.2%)	14(48.3%)	15(51.7%)
Genetic syndromes	13(7.7%)	6(46.2%)	7(53.8%)
Chronic diseases	36(21.3%)	24(66.7%)	12(33.3%)
Cause not established	12(7%)	5(41.7%)	7(58.3%)
Total	169	91(53.8%)	78(46.2%)

 Children with short stature in 11-15 years age group (n=47) had pubertal development stage I & II in 33(70.21%) followed by 10(21.28%) children with stage III and 4(8.51%) children with stage IV. None of the children had stage V.

 Most common single etiological factor for short stature was familial / genetic short stature evident in 36(21.3%) children followed by hypothyroidism in 29(17.2%) children. There were 18(10.7%) children with growth hormone deficiency, 11(6.6%) with CDGM, 8(4.7%) with primary malnutrition, 6(3.6%) with celiac disease as a cause of short stature. Diagnosis could not be made in 12 (7%) children as the parents were not willing for investigations (Table-II). Genetic syndromes as cause of short stature were present in 13 (7.7%) children. Among these, 4 children (2.4%) had unknown syndrome, 5(3%) had Turner’s syndrome, 2 children (1.2%) Laurence-Moon-Bardet-Biedl syndrome and 2(1.2%) Noonan syndrome. Chronic diseases were the cause of short stature in 36 (21.3%)(Table-III).

## Discussion

 Growth is an important objective parameter of health of a child. Short stature although not a disease per se, may be a manifestation of several diseases. The etiology of short stature ranges from normal variants like CDGM and FSS to pathological conditions like endocrine and systemic disorders. Timely assessment is very important, because medical intervention if needed will only be effective before epiphyseal fusion.

**Table-III:** Chronic Diseases as Cause of Short Stature in Children.

*Diseases*	*No. of Children*	*Percentage (%)*
Congenital heart disease (VSD)	2	1.2
Insulin-Dependent Diabetes Mellitus	16	9.5
Thalassemia	2	1.2
Bronchial Asthma	2	1.2
Rickets	2	1.2
Fanconi Anemia	2	1.2
Low birth weight	4	2.4
Addison’s disease	2	1.2
Epilepsy	4	2.4

Among 169 cases in this study, most common age group was >5 to 11 years 82(48.5%) cases. The observed male to female ratio was 1.17:1. Frequently observed causes of short stature included; FSS 36(21.3%) cases, hypothyroidism 29(17.2%), GHD 18(10.7%), IDDM 16(9.5%), CDGM 11(6.6%), primary malnutrition (3^rd^ degree) 8(4.7%) and celiac disease 6(3.6%) cases. This was followed by other rare causes.

 In this study if we categorize the causes in three main etiological groups; the most frequent as a group were endocrinological causes followed by NVSS and then nonendocrinological causes. Similar observation was made by Colacoetal^22^ in their study in Indian children and found that the endocrinological causes were the most common. On the other hand a study conducted at Combined Military Hospital Multan Pakistan,^23^ nonendocrinological diseases as a group were the most common cause (46.7%), followed by NVSS(37.4%) and endocrinological causes were the least (15.9%). Shu et al^24^ in their study showed that upto 65% cases of short stature were NVSS. The dominance of NVSS was in accordance with other international studies.^25^

 In our study GHD was seen in 18(10.7%) cases whereas it was found to be 23.4%, 22.8% and 13.9% in studies done by Moayeri et al,^18^ Zarger et al,^25^ and Awan TM et al.^26^ These values for GHD were relatively higher, probably all these studies were conducted at endocrine referral centers, therefore, problems of endocrine disturbances, especially growth hormone were relatively more. In Utah growth study,^27^ nonendocrinological causes predominate, endocrine contribution was less than 4% in children with short stature. In the same study, protein energy malnutrition and malabsorption syndrome were responsible for 10% of short stature cases and similarly 12.7% in Indian Bhadada study,^3^ but in this study 8 children (4.7%) with primary malnutrition and 6 children (3.6%) with celiac disease presented as the causes of short stature.

 The shortcomings of this study include failure to calculate and plot growth velocity which requires a regular follow-up at six months to twelve months interval, which was not possible in this cross sectional study. Secondly, it was a hospital based study where patients of specific diseases are referred. Twelve cases remained undiagnosed, because parents were not willing for investigations. The difference in frequency of various causes of short stature reported from different centers can be due to many factors like genetic, socioeconomic, nutritional and other related factors.

 It is very important to know exactly the frequency of various causes of short stature from a given population in order to differentiate normal variants of growth from individual cases of short stature who need early diagnosis and treatment. Statistics addressing frequencies of various causes of growth failure in Pakistan are not plentiful. This study may help to set a base line data in this region, so early detection of treatable causes would be helpful in a better long-term prognosis.

## Conclusions

 The endocrinological causes of short stature as a group were the most common followed by normal variants short stature and nonendocrinological causes were the least in descending order of frequency. The male to female ratio was almost equal. Larger scale, community based studies may give results which are better representative of a particular population.
